# Sensitization of Radioresistant Prostate Cancer Cells by Resveratrol Isolated from *Arachis hypogaea* Stems

**DOI:** 10.1371/journal.pone.0169204

**Published:** 2017-01-12

**Authors:** Yu-An Chen, Hsiu-Man Lien, Min-Chuan Kao, U-Ging Lo, Li-Chiung Lin, Chun-Jung Lin, Sheau-Jiun Chang, Chia-Chang Chen, Jer-Tsong Hsieh, Ho Lin, Chih-Hsin Tang, Chih-Ho Lai

**Affiliations:** 1 Graduate Institute of Basic Medical Science, School of Medicine, China Medical University, Taichung, Taiwan; 2 Research Institute of Biotechnology, Hungkuang University, Taichung, Taiwan; 3 Department of Microbiology and Immunology, Graduate Institute of Biomedical Sciences, College of Medicine, Chang Gung University, Taoyuan, Taiwan; 4 Department of Urology, University of Texas Southwestern Medical Center, Dallas, Texas, United States of America; 5 Department of Life Sciences, National Chung Hsing University, Taichung, Taiwan; 6 Department of Rehabilitation, Dachien General Hospital, Miaoli, Taiwan; 7 School of Management, Feng Chia University, Taichung, Taiwan; 8 Department of Nursing, Asia University, Taichung, Taiwan; 9 Molecular Infectious Disease Research Center, Chang Gung Memorial Hospital, Taoyuan, Taiwan; Taipei Medical University, TAIWAN

## Abstract

Resveratrol (RV, 3,4ʹ,5-trihydroxystilbene) is naturally produced by a wide variety of plants including grapes and peanuts (*Arachis hypogaea*). However, the yield of RV from peanut stem and its potential radiosensitizing effects in prostate cancer (PCa) have not been well investigated. In this study, we characterized RV in peanut stem extract (PSE) for the first time and showed that both RV and PSE dose-dependently induced cell death in DOC-2/DAB2 interactive protein (DAB2IP)-deficient PCa cells with the radioresistant phenotype. Furthermore, the combination of radiation with either RV or PSE induced the death of radioresistant PCa cells through delayed repair of radiation-induced DNA double-strand break (DSB) and prolonged G2/M arrest, which induced apoptosis. The administration of RV and PSE effectively enhanced radiation therapy in the shDAB2IP PCa xenograft mouse model. These results demonstrate the promising synergistic effect of RV and PSE combined with radiation in the treatment of radioresistant PCa.

## Introduction

Prostate cancer (PCa) is the most commonly diagnosed cancer among men in western countries, and the incidence is currently rising in Asia [1]. Radiotherapy is considered as the first-line treatment for localized PCa because of its non-invasiveness [2]. It is generally accepted that the primary therapeutic effect of ionizing radiation (IR) is the induction of DNA damage in the irradiated cells [3]. However, PCa cells often become resistant to radiation after a prolonged period of radiation therapy [4,5]. It has been reported that the DOC-2/DAB2 interactive protein (DAB2IP) is a potent suppressor of PCa tumor progression [6]. The loss of DAB2IP expression allows PCa cells to proliferate, enhances their anti-apoptotic potential [7,8], and develops a cancer stemness phenotype [9], which confers resistance to radiation-induced apoptosis [10]. Therefore, the development of potent therapeutic agents that increase the sensitivity of malignant prostate cells to radiation is urgently required.

Resveratrol (RV, 3,4ʹ,5-trihydroxystilbene) is a polyphenolic compound produced by a wide variety of plants including grapes, peanuts (*Arachis hypogaea*), and soy [11]. Previous studies have demonstrated that RV possesses several biological activities including antioxidant, anti-inflammatory, and anticancer [12–15]. The anticancer property of RV has been investigated in leukemia, lung and breast cancers, colon carcinoma, hepatoma, and PCa [16–22]. Although studies have suggested that RV could be used as a radiosensitizer in cancer therapy, its therapeutic potential requires comprehensive exploration.

Immature peanut kernels have been reported to contain high levels of RV, but the levels decrease as the plants mature [23]. All parts of the peanut plant including the shell, skin, and even root have been shown to contain RV [24]. However, RV occurs at very low levels in edible peanuts and peanut products and has not been isolated from the stems [25].

In this study, we characterized RV in peanut stem extract (PSE) for the first time and evaluated its therapeutic effects in radioresistant PCa. We also investigated the molecular mechanism underlying the radiosensitization induced by PSE and RV in PCa. Our results demonstrated that PSE contains a high content of RV, which could be developed as a potent therapeutic agent for overcoming radioresistance in PCa.

## Materials and Methods

### Antibodies and reagents

Antibody specific for 53BP1 and β-actin were purchased from Santa Cruz Biotechnology (Santa Cruz, CA). Antibodies against cleaved PARP (Asp214), cleaved Caspase3 (Asp175) and Caspase 9 were purchased from Proteintech (Chicago, IL). Antibodies against phospho-ATM, ATM, phospho-checkpoint kinase 2 (CHK2), CHK2, phospho-p53, and p53were purchased from Cell Signaling (Danvers, MA). Anti-phospho-γ-H2AX (Ser139) antibody was purchased from Millipore (Billerica, MA). Alexa Fluor 488–conjugated goat anti-mouse IgG, and Alexa Fluor 594–conjugated goat anti-rabbit IgG, and 4',6-diamidino-2-phenylindole (DAPI) were purchased from Molecular Probes (Invitrogen, Carlsbad, CA). All other reagents were purchased from Sigma-Aldrich (St. Louis, MO).

### Cell culture

DAB2IP knockdown cell lines (LAPC4-KD and PC3-KD) were constructed by using the shRNA system (pGIPZ-lentiviral-shRNAmir from Open Biosystems, Huntsville, AL) and selected under puromycin. The cell lines were kindly provided by Dr. Jer-Tsong Hsieh (Department of Urology, University of Texas Southwestern Medical Center) [26]. LAPC4-KD cells were maintained in Iscove's Modified Dulbecco's Medium (IMDM) (Gibco, Grand Island, NY) supplemented with 5% fetal bovine serum (FBS) (Hyclone, Logan, UT). PC3-KD cells were maintained in RPMI1640 supplemented with 5% FBS. The cells were cultured in a humidified atmosphere containing 5% CO_2_ [27].

### Isolation and characterization of RV from peanut stems

The peanut stems of *Arachis hypogaea* Linn. Tainan no. 12 were collected by Yushen Biotechnology (Taichung, Taiwan). The geographical coordinate for the collection site was 23°57'55.1"N 120°42'31.9"E. No permission was required for the area. The prepared stems were washed with deionized water, and dehydrated at 40–45°C in a forced-air oven. For each extraction, 5 g of the dehydrated peanut stems were deposited into a 10-ml centrifuge tube and extracted with 70% (v/v) ethanol following incubation at 50°C for 1 h. The tubes were centrifuged at 8000 *g* at 20°C for 15 min, and the supernatants were membrane-filtered (0.45 μm) for HPLC analysis. The filtrate was evaporated under reduced pressure at a temperature not exceeding 35°C to obtain the crude extract, which was subsequently stored at -20°C until analyzed. The detection and quantification of RV in the extract was performed by using a reversed-phase HPLC method using a Waters e2695 (Waters Separations Module, MI, USA) equipped with a quaternary pump, autosampler, and 2998 photodiode array detector (PDA) [28]. In addition, the Waters Empower software for peak identification and integration was used for the data HPLC data analysis. A LiChrospher column C-18 with a 250 × 4.6 mm i.d. 5 μm (Merck) was run at a temperature under 40°C with flow rate, sample injection volume, and a detection wavelength of 10 mL/min, 20 μL, and 318 nm, respectively. The mobile phase was trifluoroacetic acid (Alfa Aesar, HPLC grade) 0.1% in water (A) and acetonitrile (ACN, B, Merck, HPLC grade), with a gradient schedule of B 15–21% for 45 min and B in A, 21–100% from 45–50 min. All samples and standards were filtered using membranes with a pore size of 0.45-μm (Millipore) and injected into the HPLC system in triplicate. The retention time and ultraviolet (UV spectra of the RV were compared to those of the high-purity commercial standard (Sigma-Aldrich).

### Cell viability assay

The mitochondrial respiration-dependent 3-(4,5-dimethylthiazol-2-yl)-2,5-diphenyl tetrazolium bromide (MTT) assay was used to determine the cytotoxicity of PSE on the growth of PCa cells. Briefly, cells were treated with various concentrations of PSE. Relative cell number was quantified by a spectrophotometer (BioRad, Hercules, CA) at the wavelength of 570 nm [29].

### Ionizing radiation

LAPC4-KD cells and nude mice were irradiated at room temperature in ambient air using the Faxitron RX-650 irradiator (Faxitron X-ray, Wheeling, IL) at the indicated doses described in each experiment [27].

### Clonogenic survival assay

The surviving fraction (SF) of each treatment in cells was assessed by following our previous report with a slight modification [10]. Briefly, LAPC4-KD cells were plated for 16 h to allow cell attachment followed by increasing doses of IR alone (2–6 Gy), IR combined with RV (25 μg/ml) or PSE (500 μg/ml). After 10-day incubation, the colonies were fixed with 4% formaldehyde in PBS and stained with 0.05% crystal violet in PBS. The number of surviving colonies (defined as a colony with > 50 cells) was counted as (mean colony counts) / (cells inoculated) / (plating efficiency) and the plating efficiency was defined as (mean colony counts) / (cells inoculated for un-irradiated controls). The data are presented as the mean ± SEM of three independent experiments. The curve S = e^–(αD+ßD2)^ was fitted to the experimental data using a least squares fitting algorithm. Linear Quadratic (LQ) analysis was subjected for radiation surviving curve and calculated with the program Sigma Plot 11.0 (Systat Software, San Jose, CA).

### Cell cycle analysis

LAPC4-KD cells were treated with IR (2 Gy), or peanut stem extracts (500 μg/ml) combined with IR. Cells were then incubated at 37°C for 24 h and 48 h. The treated cells were harvested and fixed with ice-cold 70% ethanol for 1 h and stained with 20 μg/ml propidium iodide (Sigma-Aldrich) containing 1 mg/ml RNase (Sigma-Aldrich) for 1 h. The stained cells were determined by FACScalibur flow cytometer (Becton-Dickinson, San Jose, CA) and the data were analyzed using Cell Quest software WinMDI (Verity Software House, Topsham, Me) as described previously [30].

### Western blot analysis

LAPC4-KD cells treated with resveratrol or peanut stem extracts combined with IR for 24 h were prepared for western blot analysis. The samples were resolved by 10% SDS-PAGE and transferred onto polyvinylidene difluoride membranes (Millipore) as described previously [31]. Briefly, membranes were probed with primary antibodies as indicated and then incubated with horseradish peroxidase−conjugated secondary antibody (Santa Cruz). The proteins of interest were detected using the ECL Western Blotting Detection Reagents (GE Healthcare, Piscataway, NJ) and visualized using X-ray film (Kodak, Rochester, NY). The signal intensity of each protein was quantified with the Image J software (National Institute of Health, Bethesda, MD).

### Immunofluorescence staining

LAPC4-KD cells (1 × 10^6^ cells/well) were plated on glass coverslips in 6-well plates. After treatment, cells were washed and fixed for immunofluorescence staining as described previously [32]. Briefly, the prepared samples were probed with phospho-histone-γ-H2AX antibody (Ser139) (Millipore) and p53-binding protein 1 (53BP1) antibody (Santa Cruz) for 2h followed by incubated with Alexa Fluor 488-conjugated anti-mouse antibody and Alexa Fluor 594-conjugated anti-rabbit antibody (Invitrogen) for 1 h. Nuclei were counterstained with 4’,6-diamidino-2-phenylindole (DAPI) (0.2 μg/ml) for 10 min. The stained cells were analyzed under a fluorescence microscope (Carl Zeiss, Göttingen, Germany) with a 63× objective (oil immersion, aperture 1.3).

### Animal study

Male nude mice (BALB/cAnN.Cg-Foxnlnu/CrlNarl, National Laboratory Animal Center) with 6-weeks old were used in this study. The mice were treated in accordance with the Animal Care and Use Guidelines for Chang Gung University. A suspension of LAPC4 shDAB2IP (2 × 10^6^) cells mixed with 50% Matrigel (BD Biosciences, Bedford, MA) in 0.1 ml was injected subcutaneously into the right posterior flanks of the mice as described previously [27]. Mice were divided into four groups (6 animals/group), including untreated control, radiation alone, combined treatment of resveratrol (5 mg/kg) or peanut stem extracts (250 mg/kg) with irradiation. After each administration, the mice were treated with a total dose of 12 Gy delivered in 3 fractions using the Faxitron RX-650 irradiator (Faxitron X-ray, Wheeling, IL) on day 0, 3 and 7. Tumors were measured 3 times per week using a Vernier caliper. Results were evaluated with the formula: volume = 0.5236 × length × width × height [33].

### Ethics statement

This study was carried out in accordance with the Institutional Animal Care Use Committee, Chang Gung University. Mice were housed in pathogen-free microisolator-type cages in a temperature (22 ± 1°C) and humidity (50 ± 5%) controlled room with a 12 h day/night cycle. Mice were free-fed with 5053 Laboratory Rodent diet (LabDiet, St. Louis, MO) and RO water. On irradiation and gavage feeding days, mice were observed every 2 h for 12 h to check for unexpected acute reactions to dosage. Clinical signs (i.e. over 15–20% body weight loss, loss of appetite for 24–36 h, loss of coordination etc.) were observed two times per day during the experimental protocol. Mice with exhibiting obvious signs of distress were taken and humanely euthanized. Animals were euthanized (CO_2_ inhalation for 10 minutes followed by cervical dislocation to ensure euthanasia) by following University of Chang Gung Animal Care and Use Committee standard operating protocols. All efforts were made to minimize the suffering from experimental procedure. The experimental protocols were approved by the Institutional Animal Care Use Committee, Chang Gung University (CGU15-076).

### Immunohistochemistry (IHC) analysis

Tissue specimens from mice were formalin-fixed then subjected to IHC staining as described previously [27,34]. Briefly, the tissue sections were de-waxed and rehydrated. The sections were stained with monoclonal antibodies against cleaved PARP and cleaved caspase3 (Cell Signaling) for 24 h at 4°C. After washing, the samples were probed with peroxidase-labeled goat anti-rabbit secondary antibody (Epitomics, Burlinggame, CA) and detected with an ABC kit (Vector Laboratories, Burlingame, CA).

### Statistical analysis

Statistical analyses for the data between two groups were determined using Student *t*-test. Statistics analysis comparisons of more than two groups were evaluated using two-way analysis of variance (ANOVA). *P* < 0.05 was considered statistically significant.

## Results

### Characterization of RV isolated from peanut stems

**Fig 1:**
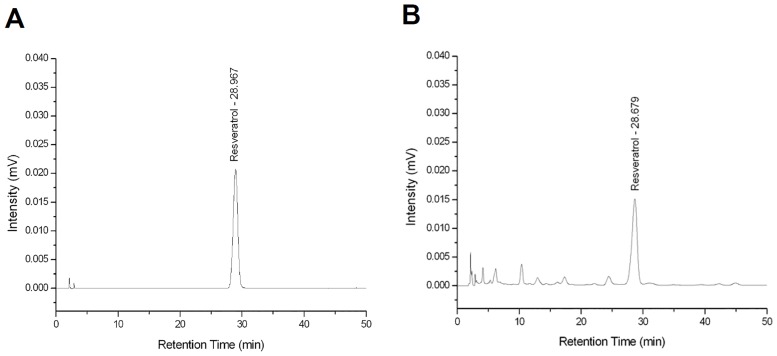
High-performance liquid chromatography (HPLC) chromatograms of resveratrol (RV). The tested samples (A) standard RV samples, and (B) RV isolated from peanut stem extract (PSE) of *Arachis hypogaea*.

RV is an active polyphenolic compound with anticancer effects and has been isolated from peanuts and peanut products [16–18,35]. However, there have no reports indicated that RV can be isolated from peanut stems [25]. To obtain sufficient amounts of RV, the PSE of *Arachis hypogaea* was prepared, and the RV content was subsequently analyzed using high-performance liquid chromatography (HPLC) as described in the Materials and Methods section. The HPLC analysis showed that RV was the major component of the PSE (Fig 1), which had a maximum absorbance, retention time, and RV content of 318 nm, 28.9 min, and 0.408 mg/g, respectively.

### RV and PSE inhibit radioresistant PCa cell proliferation

**Fig 2:**
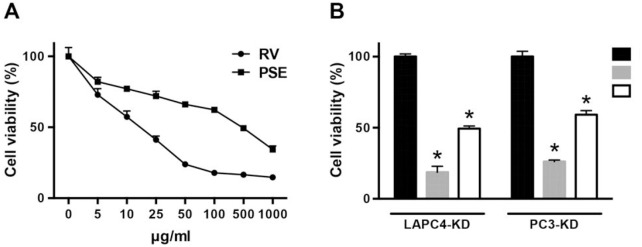
Resveratrol (RV) and peanut stem extract (PSE) inhibit proliferation of radioresistant prostate cancer. (A) LAPC4-KD were treated with RV or PSE at indicated concentrations (0–1000 μg/mL) for 48 h. (B) LAPC4-KD and PC3-KD cells were treated with RV or PSE (25 or 500 μg/mL, respectively) for 48 h. Cell viability was assessed using the 3-(4,5-dimethylthiazol-2-yl)- 2,5-diphenyltetrazolium bromide (MTT) assay. Statistical significance was evaluated using Student’s *t*-test (*, *P* < 0.01).

Because the PSE contained a high RV content, we first examined whether PSE inhibited the growth of human PCa cells. Our previous study demonstrated that the knockdown of DAB2IP (shDAB2IP) in PCa cells exhibited a radioresistant phenotype [10]. Therefore, the DAB2IP knockdown (KD) radioresistant cell lines, LAPC4-KD and PC3-KD, were used in this study. The cells were cultured with various concentrations of RV or PSE for 48 h. As shown in Fig 2A, RV and PSE effectively inhibited LAPC4-KD cell proliferation dose-dependently with a half-maximal inhibitory concentration (IC_50_) of approximately 25 and 500 μg/mL, respectively. In addition, treatment of LAPC4-KD cells with RV and PSE significantly inhibited cell proliferation compared to the mock-treated control cells (Fig 2B). Similarly, RV and PSE exhibited a significant cytotoxic effect against the PC3-KD PCa cell line, which possesses a radioresistant phenotype. These results indicate that PSE contains a high RV content, which remarkably inhibited the proliferation of radioresistant PCa cells.

### PSE enhances sensitivity of PCa cells to IR

**Fig 3:**
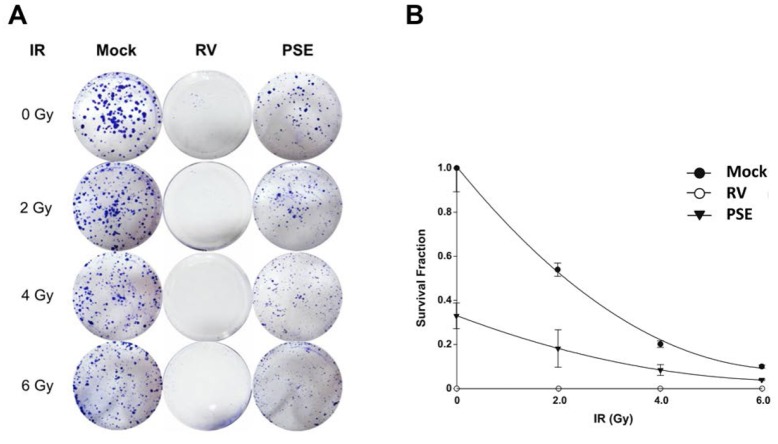
Resveratrol (RV) and peanut stem extract (PSE) increase radiosensitivity of prostate cancer cells. LAPC4-KD cells were treated with irradiation (IR, 0–6 Gy) alone or combined with RV or PSE (25 or 500 μg/mL, respectively). After 10-day incubation, survival cell colonies were (A) stained with crystal violet and (B) assessed using clonogenic assays as described in the methods section.

We determined whether RV and PSE increased the sensitivity of PCa cells to IR. The LAPC4-KD cells were treated with IR (2–6 Gy) alone or in combination with either RV or PSE (25 or 500 μg/mL, respectively), and then analyzed using a clonogenic assay. As shown in Fig 3, PSE synergistically enhanced the radiosensitivity of LAPC4-KD cells with increasing IR doses. Furthermore, the combined treatment with RV and IR enhanced the susceptibility of the cells to IR compared to IR treatment alone. These results demonstrate that PSE and RV effectively enhanced the IR-induced death of radioresistant PCa cells.

### PSE induces apoptosis by cell cycle arrest

**Fig 4:**
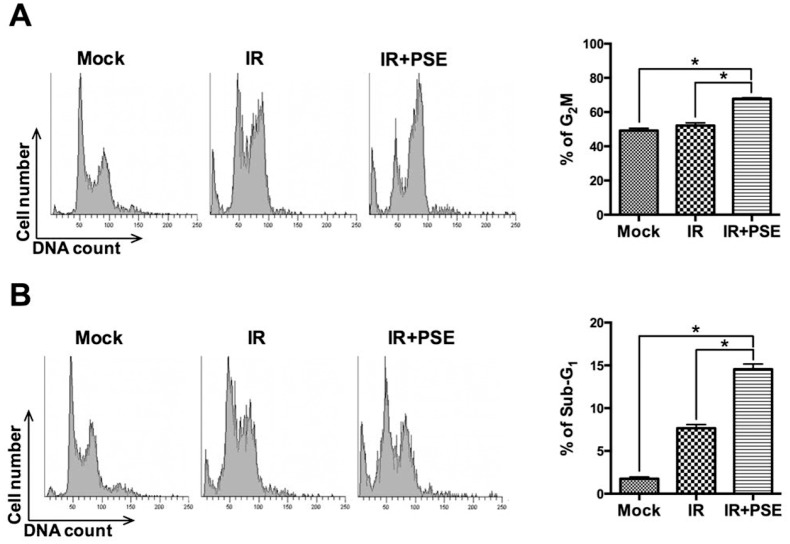
Peanut stem extract (PSE) induces cell cycle arrest followed by apoptosis of radioresistant prostate cancer cells. LAPC4-KD cells were untreated (mock) or exposed to IR (2Gy) alone or combined with PSE (500 μg/mL) and incubated for (A) 24 and (B) 48 h. Cell cycle distribution based on DNA content was analyzed using flow cytometry. Percentages of cells in G2/M and sub-G1 phases were calculated and plotted as intensity bars in right panels.

We further assessed whether PSE induced cell cycle arrest in radioresistant PCa cells, by treating LAPC4-KD cells with IR (2 Gy) alone or in combination with PSE (500 μg/mL) for 24 h followed by cell cycle analysis. The results showed that treatment of cells with IR alone slightly induced G2/M arrest compared to the mock-treated control cells (Fig 4A); however, combination treatment with IR and PSE markedly arrested the cell cycle at the G2/M phase. Then, we determined whether the PSE-induced LAPC4-KD cell death was mediated by an apoptotic mechanism. The cells were irradiated with 2 Gy alone or combined with PSE (500 μg/mL) for 48 h and then subjected to cell cycle analysis. As shown in Fig 4B, the sub-G1 cell population, after IR alone treatment, slightly increased compared to mock-treated control group. However, a large proportion of sub-G1 cells was observed following co-treatment with IR and PSE. In addition, the expression levels of apoptotic molecules including cleaved PARP, caspase 3, and caspase 9 were significantly elevated in cells treated with the combination of PSE and IR when compared to that treated with IR alone (S1 Fig). These results indicate that PSE remarkably enhanced IR-induced apoptosis in radioresistant PCa cells via cell cycle arrest.

### Double-Strand Break (DSB) pathway mediates PSE-induced enhancement of radiosensitivity

**Fig 5:**
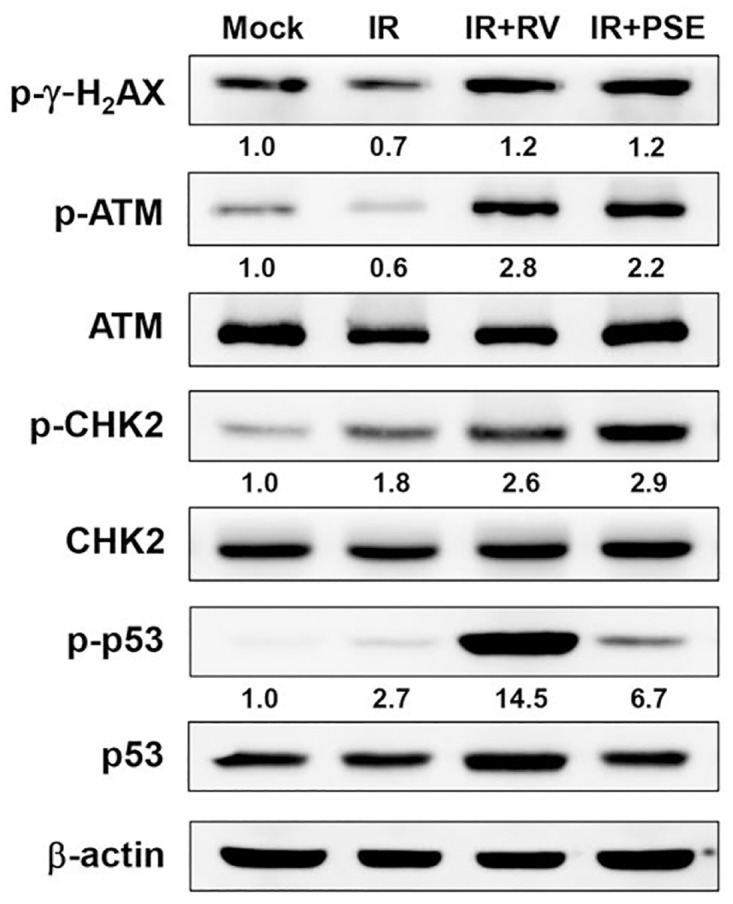
Resveratrol (RV) and peanut stem extract (PSE) enhance irradiation (IR)-induces double-strand breaks (DSBs) in radioresistant prostate cancer cells. Protein expression levels of target molecules in LAPC4-KD cells that were untreated (mock), exposed to IR (2Gy) alone, or in combination with RV or PSE (25 or 500 μg/mL, respectively). Representative western blot results from one of three independent experiments are shown. β-actin was the loading control. Expression level of each protein was quantified using signal intensity and indicated below each lane.

To delineate the mechanism underlying the PSE-induced cell cycle arrest and apoptosis in LAPC4-KD cells, the expression levels of key molecules involved in cell death were further examined. As shown in Fig 5, the expressions of the phosphorylated histone 2A family member X (p-γ-H_2_AX), p-ataxia telangiectasia mutated (ATM), p-checkpoint kinase 2 (CHK2), and p-p53 were sustained at a low level in cells treated with IR alone. However, the expression of these phospho-proteins increased following co-treatment with IR and either RV or PSE compared with the mock-treated control group. The effect of PSE on the induction of DNA damage was then assessed by visualizing the co-localized foci of p-γ-H_2_AX and the tumor suppressor p53 binding protein 1 (53BP1) using immunofluorescence microscopy (S2A Fig). Our data showed that the co-localization of γ-H2AX and 53BP1 foci significantly increased in the cells co-treated with IR and PSE compared to that in cells treated with IR alone (S2B Fig). These results demonstrate that PSE effectively enhanced the IR-induced DSB and prolonged the DSB repair kinetics of the PCa cells.

### PSE synergistically enhances IR-suppressed PCa growth *in vivo*

**Fig 6:**
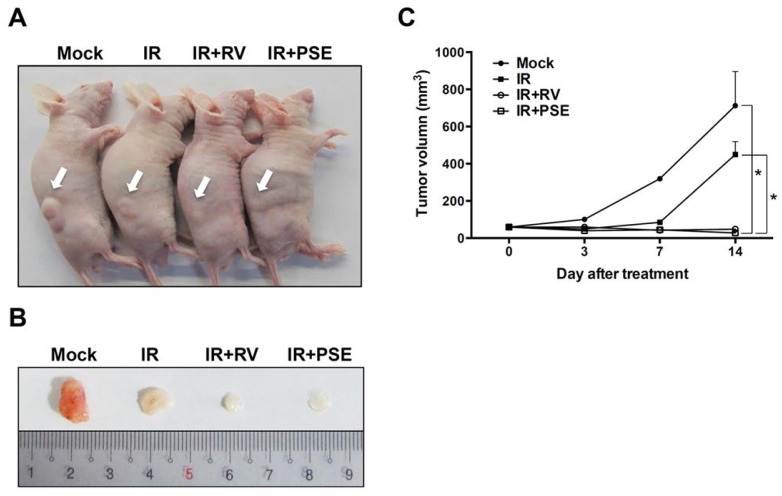
Resveratrol (RV) and peanut stem extract (PSE) significantly enhance irradiation (IR)-suppression of prostate cancer growth *in vivo*. (A) Mice with xenograft tumors were divided into four groups and were treated with vehicle (mock, control) or IR alone or combined with RV or PSE (5 or 250 mg/kg, respectively). Arrows indicated tumor that was grown in the posterior flanks of mice. (B) Treatments were administered on day 0, 3, 7, and 14. After euthanasia, tumors were excised from mice. Scales shown in images are in centimeter. (C) Tumor volume was calculated, and data are means ± standard deviations; *, *P* < 0.01 compared to each group.

**Fig 7:**
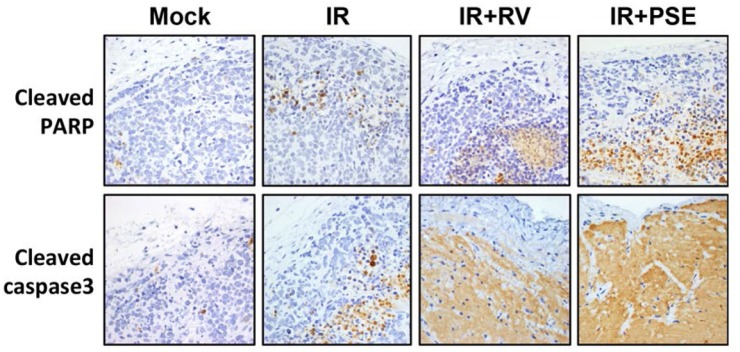
Representative immunohistochemical (IHC) staining patterns in xenograft prostate cancer tissue. IHC of paraffin sections shows staining with specific antibodies against cleaved poly adenosine diphosphate (ADP) ribose polymerase (PARP) and caspase 3. Images were photographed at 200× magnification.

To further evaluate the efficiency of the PSE-induced radiosensitization of PCa cells, we established a tumor xenograft mouse model. The tumors were mock-treated or exposed to IR alone or in combination with either RV or PSE (5 or 250 mg/kg, respectively). As shown in Fig 6, the mice treated with IR alone showed tumor growth that was only slightly reduced compared to that of the mock-treated control. However, the combination of IR with RV or PSE dramatically inhibited tumor growth. Then, we analyzed the expression of apoptotic markers in the tumor tissues and our data showed that the expression levels of cleaved poly adenosine diphosphate (ADP) ribose polymerase (PARP) and caspase 3 increased in tumors co-treated with IR and either RV or PSE (Fig 7). These results demonstrate that the PSE-enhanced IR-induced cell death was mediated by an apoptotic mechanism that was similar to that of RV.

## Discussion

The increasing incidence of radio- and chemo-resistance in the treatment of PCa requires the urgent development of potent therapeutic agents to overcome refractory PCa. RV, a plant-derived polyphenolic phytoalexin, potentiates the effects of radiation and chemotherapeutic agents in PCa cells [36,37]. RV can be isolated from a wide variety of plants including grapes, peanuts, and soy. However, the content of RV is varied among the plants. It has been reported that red grape juice and grape skin contained 0.5 and 10 μg/g of RV, respectively [38]. Cranberries contains an RV content of 0.278 μg/g [39].

Although RV can be isolated from a wide range of fruits and other plants including peanuts, its content is low in mature peanuts [23]. Previous studies have reported that roasted peanuts and peanut butter contain an RV content of 0.05 and 0.324 μg/g, respectively [25]. Peanuts from various markets were shown to contain 0.03–0.14 μg/g of RV [40]. However, there have been no reports on the isolation of RV from peanut stems. In this study, we isolated RV from peanut stems, which were the inedible and discarded peanut parts. We reported that the RV content of the PSE was 0.408 mg/g, which was much higher than that of commercial peanuts and their products. Therefore, to the best of our knowledge, our study is the first to demonstrate the isolation of RV from peanut stems, thereby indicating that this could be a potential source of large amounts of RV. The results of this study also illustrate that the regeneration and reuse of plant parts could be beneficial to humans during periods of food exhaustion or scarcity.

The anticancer property of RV has been investigated in several types of cancer cells. For instance, RV was able to reduce migration and invasion through the inhibition of PI3K/Akt signaling pathway in MDA-MB 435 cells [41]. RV increased apoptosis by inhibiting phosphodiesterase 4 (PDE4) activity in a colorectal cancer cell line, HCT116 [42]. In addition, RV induced cell death in PCa cell lines, PC3 and DU145, by inhibiting AKT/mTOR and activating AMPK pathway [43]. Moreover, RV can enhance CD95 expression in HL60 human leukemia cell line, leading to cell apoptosis [44].

DAB2IP plays an important role in the regulation of cell proliferation, survival, and apoptosis by phosphoinositide 3-kinase (PI3K)-AKT inactivation mediated by the apoptosis signal-regulating kinase 1 (ASK1) activation pathways [7,26]. Decreased DAB2IP expression is found in PCa with increased risk of tumor metastasis [45]. Our previous studies demonstrated that the downregulation of DAB2IP in PCa conferred the cells with resistance to stress-induce apoptosis [7] and markedly increased their resistance to radiation [10]. Additionally, low DAB2IP expression levels enhanced the accelerated process of repair by DSB, a G2/M checkpoint, which is a functional system for evading apoptosis [10]. Therefore, the inhibition of the DAB2IP-regulatory pathway could be a useful therapeutic strategy for the treatment of radioresistant PCa. In this study, we showed that the radioresistance of shDAB2IP PCa cells was overcome by co-treatment with IR and either RV or PSE (Fig 3). The mechanisms underlying the radiosensitization induced by RV or PSE in DAB2IP-deficient PCa cells were mediated by DSB, cell cycle arrest at the G2/M phase, and increased sub-G1 population, which specifically increased the cell apoptosis (Fig 4). Therefore, our investigations demonstrated that RV or PSE combined with radiation induced the synergistic death of DAB2IP-deficient PCa cells.

Increased DSB is accompanied by the formation of histone γ-H2AX molecules in the cell nuclei [46]. Changes in ATM expression play a crucial role in prostate tumorigenesis [47]. Additionally, triggering checkpoint responses to radiation is one of the major barriers to preventing carcinogenesis [48]. In this study, combination treatment with IR and RV or PSE remarkably elevated the expressions of phospho-proteins including p-γ-H2AX, p-ATM, p-CHK2, and p-p53 (Fig 5). Moreover, the DSB repair kinetics was prolonged in cells co-treated with IR and PSE (S2 Fig). Our results clearly demonstrated that the mechanisms underlying radiosensitization induced by RV and PSE in PCa cells involve the regulation ATM-dependent checkpoint responses.

RV has been extensively explored for potential inhibitory effects against PCa. Previous studies have been performed using a considerable number of cell-based and few animal experiments, but there have been no clinical trials in human subjects [14,37]. Several PCa cell lines have been used to investigate the anticancer activity of RV including PC-3 (androgen receptor-negative), C4-2, and CWR22RV1 (both androgen-independent) cells. Treatment of PC-3 cells with low-dose radiation (2Gy) showed resistance to IR [49]. However, high-dose radiation (6-8Gy) was highly toxic to the PC3 cells, which may not justify its clinical usefulness. In this study, the cell assay models used were DAB2IP-deficient PCa cells (LAPC4-KD and PC3-KD), which have a characteristic radioresistant phenotype [10]. Treatment of DAB2IP-deficient PCa cells with high-dose radiation even at 10 Gy, showed no significant apoptosis [10]. In addition, downregulation of DAB2IP increased the EMT and enriched the cancer stem cell population of the PCa cells [50]. This established line of evidence indicates that DAB2IP knockdown-induced radioresistant PCa cells could be successfully used to evaluate the mechanism underlying the potential radiosensitization induced by PSE and RV. In addition, the PCa xenograft animal model was used to confirm the potent radiosensitizing activity of PSE and RV. The results of this study support the use of PSE and RV as therapies for radioresistant PCa, which precisely reflect their clinical relevance and usefulness. However, there have been no human clinical trials on the therapeutic effects of PSE and RV in PCa and therefore, further investigations in humans would be required.

## Conclusion

This study demonstrated that PSE contains a high RV content that can enhance its radiosensitization effects, which is likely mediated by the attenuation of DSB repair, long-term cell-cycle arrest in the G2/M phase, and activation of the apoptotic pathway. Finally, our results indicate that the RV isolated from the peanut stems may have the potential to be developed as a new therapeutic agent for co-administration with radiation to overcome radioresistant PCa.

## Supporting Information

S1 Fig Peanut stem extract (PSE) induces apoptosis of radioresistant prostate cancer cells. LAPC4-KD cells were untreated (mock) or exposed to IR (2Gy) alone or combined with PSE (500 μg/mL) and incubated for 48 h. The expression levels of PARP, caspase 3, and caspase 9 were analyzed. Representative western blot results from one of three independent experiments were shown. β-Actin expression was used as the loading control.
(PDF)

S2 Fig Double-strand break (DSB)-associated molecules are evident following treatment with PSE. (A) LAPC4-KD cells were untreated (mock) or treated with irradiation (IR, 2Gy) alone or in combination of with peanut stem extract (PSE, 500 μg/mL). Cells were cultured for 24 h and the foci of phospho-γ-H_2_AX (green) and 53BP1 (red) were detected using immunostaining. Scale bar, 10 μm. (B) Number of co-localized foci formed was determined. *, *P* < 0.01 was considered significant. H_2_AX, phosphorylated histone 2A family member X.
(PDF)
