# Supplementary figures and images for: Sensitization of Radioresistant Prostate Cancer Cells by Resveratrol Isolated from Arachis hypogaea Stems
Source: PLoS One. 2017 Jan 12;12(1):e0169204. doi: 10.1371/journal.pone.0169204 (PMC5231355; doi:10.1371/journal.pone.0169204)

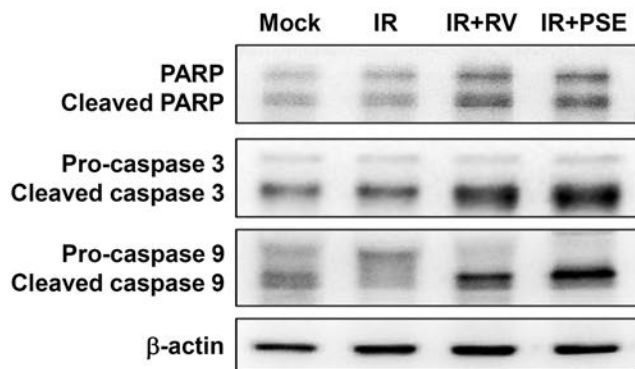

Supplement: S1 Fig — LAPC4-KD cells were untreated (mock) or exposed to IR (2Gy) alone or combined with PSE (500 μg/mL) and incubated for 48 h. The expression levels of PARP, caspase 3, and caspase 9 were analyzed. Representative western blot results from one of three independent experiments were shown. β-Actin expression was used as the loading control. (PDF) [file pone.0169204.s001.pdf]

**A**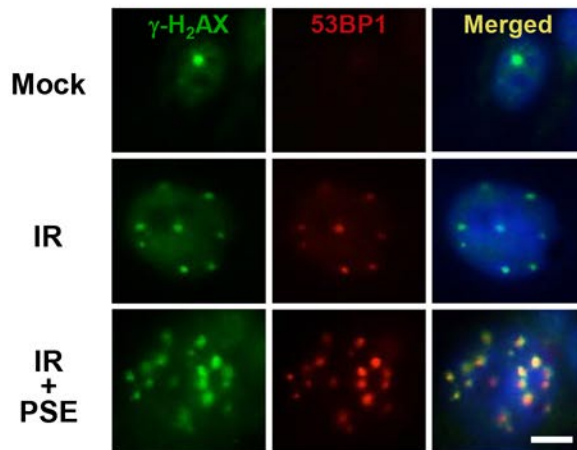**B**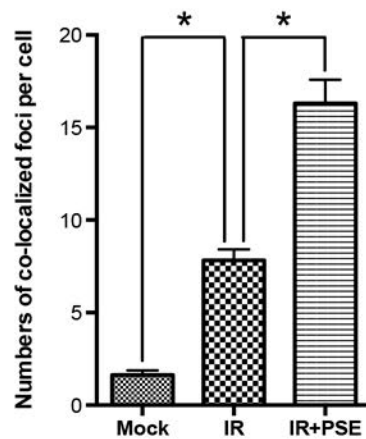

Supplement: S2 Fig — (A) LAPC4-KD cells were untreated (mock) or treated with irradiation (IR, 2Gy) alone or in combination of with peanut stem extract (PSE, 500 μg/mL). Cells were cultured for 24 h and the foci of phospho-γ-H2AX (green) and 53BP1 (red) were detected using immunostaining. Scale bar, 10 μm. (B) Number of co-localized foci formed was determined. *, P < 0.01 was considered significant. H2AX, phosphorylated histone 2A family member X. (PDF) [file pone.0169204.s002.pdf]
